# Zonulin Regulates Intestinal Permeability and Facilitates Enteric Bacteria Permeation in Coronary Artery Disease

**DOI:** 10.1038/srep29142

**Published:** 2016-06-29

**Authors:** Chuanwei Li, Min Gao, Wen Zhang, Caiyu Chen, Faying Zhou, Zhangxu Hu, Chunyu Zeng

**Affiliations:** 1 Department of Cardiology, Daping hospital, The Third Military Medical University, Chongqing, P. R. China; 2 Department of Biochemistry and Molecular Biology, The Third Military Medical University, Chongqing, P. R. China; 3 Department of respiration, Xinqiao hospital, The Third Military Medical University, Chongqing, P. R. China; 4 Department of Pediatric, Daping hospital, The Third Military Medical University, Chongqing, P. R. China

## Abstract

Several studies have reported an association between enteric bacteria and atherosclerosis. Bacterial 16S ribosomal RNA (rRNA) gene belong to *Enterobacteriaceae* have been detected in atherosclerotic plaques. How intestinal bacteria go into blood is not known. Zonulin reversibly modulate intestinal permeability (IP), the circulating zonulin levels were increased in diabetes, obesity, all of which are risk factors for atherosclerosis. It is unclear whether the circulating zonulin levels were changed in coronary artery disease (CAD) patients and modulate IP. The 16S rRNA gene of bacteria in blood sample was checked by 454 pyrosequencing. The zonulin levels were determined by enzyme-linked immunosorbent assay (ELISA) methods. The distribution of zonulin was detected by confocal immunofluorescence microscopy. Bacteria and Caco-2 cell surface micro-structure were checked by transmission electron microscopy. A high diversity of bacterial 16S rRNA gene can be detected in samples from CAD patients, most of them (99.4%) belong to *Enterobacteriaceaes*, eg. *Rahnella*. The plasma zonulin levels were significantly higher in CAD patients. *Pseudomonas fluorescens* exposure significantly increased zonulin expression and decreased IP in a time dependent manner. The elevated zonulin increase IP and may facilitate enteric translocation by disassembling the tight junctions, which might explain the observed high diversity of bacterial 16S rRNA genes in blood samples.

Inspite of great advances in the prevention and treatment of CAD, it remains to be a major cause of death worldwide^1^. The occurrence and development of CAD involves multiple factors of which inflammation activation plays an important role in the pathogenesis of atherosclerotic CAD. It is known that immune cells are not only involved into the pathogenesis of atherosclerosis, but also the major factor in initiating plaque vulnerability that subsequently leads to acute coronary syndrome^2^. Besides immune cells, infectious agents have gained a growing research interest in recent decades. Epidemiological and experimental studies have shown a linkage between CAD and several pathogens, including *Chlamydia pneumoniae*, *Helicobacter*, and *Cytomegalovirus*^3^,^4^,^5^. Intestinal microbiota comprise of trillions of typically non-pathogenic commensal organisms. However, several studies have shown that intestinal bacteria could influence cholesterol metabolism by gut flora-dependent metabolism of dietary phosphatidylcholine or carnitine and accelerate atherosclerosis^5^,^6^,^7^. There is evidence that bacterial 16S rRNA genes can be detected in as high as 95% of atherosclerotic plaque biopsies, among which *Enterobacteriacea* (e.g. *Serratia sp.* and *Klebsiella sp.*) are the most frequently found family, indicating that, besides an indirect effect, bacteria themselves might be directly engaged in the pathogenesis of CAD^8^.

The intestines harbor the largest number of bacterial colony in the body, which by far vastly outnumber the host cells in the body. However, how intestinal bacteria get into the blood and reside in atherosclerotic plaques is not known. Intestinal epithelia forms a continuous barrier between the intestinal microbiota and the host. Intestinal intercellular tight junctions, which determine the IP and control the entrance of bacteria from intestine to host blood, are the key structures. As the major component of intercellular tight junction, zonulin, a recently discovered protein, originally found in the eukaryotic counterpart of *Vibrio cholerae* zonula occludens toxin, was identified as the major factor determining the degree of IP^9^. Recent researches revealed that circulating zonulin levels are significantly elevated in patients with diabetes, polycystic ovary syndrome, obesity, nonalcoholic fatty liver disease, all of which are regarded as traditional risk factors of atherosclerosis^10^,^11^,^12^. We hypothesize therefore, that zonulin might be engaged in the pathogenesis of CAD by controlling IP and facilitate intestinal bacteria translocation to the host blood. Our present study confirmed the presence of high diversity bacteria in blood samples from CAD patients by 16S rRNA gene amplification, most of them (99.4%) belong to *Enterobacteriaceaes*. The plasma zonulin levels were significantly higher in CAD patients as compared with control. Adding the *Pseudomonas fluorescens* bacteria to the upper medium of the transwell assay significantly decreased the transepithelial electrical resistance (TEER) of Caco-2 cell monolayer in a time dependent manner. Transmission electron microscopy (TEM) revealed that coccus-shaped bacteria were entangled in the Caco-2 cell monolayer and may result in penetration by disassembling the intestinal tight junctions.

## Results

### Analysis of 16S rRNA gene sequence segments

This study enrolled 16 patients suspected with CAD, who were taking the standard medicines for CAD treatment, eg. aspirin, statins, without antibiotics. They were categorized into two groups (CAD group and non-CAD group) according to the angiography results. Demographic data of the two study groups are presented in Supplementary Table 1. There were no significant differences in terms of age, sex, diabetes or biochemical parameters. The DNA were extracted from the blood samples and mixed together with equal volume; then further analyzed by detection of the 16S rRNA gene sequences. After discarding the incomplete sequences, high-quality 16S rRNA gene sequences in the CAD group (9,203) and non-CAD group (9,064) were further analyzed, most of its distribution range was 541–561 bp. Sequences were assigned to species-level operational taxonomic units (OTUs) using a 99% pairwise-identity cutoff. The classification, sequence similarity of lower than 99% were identified as no rank.

**Figure 1:**
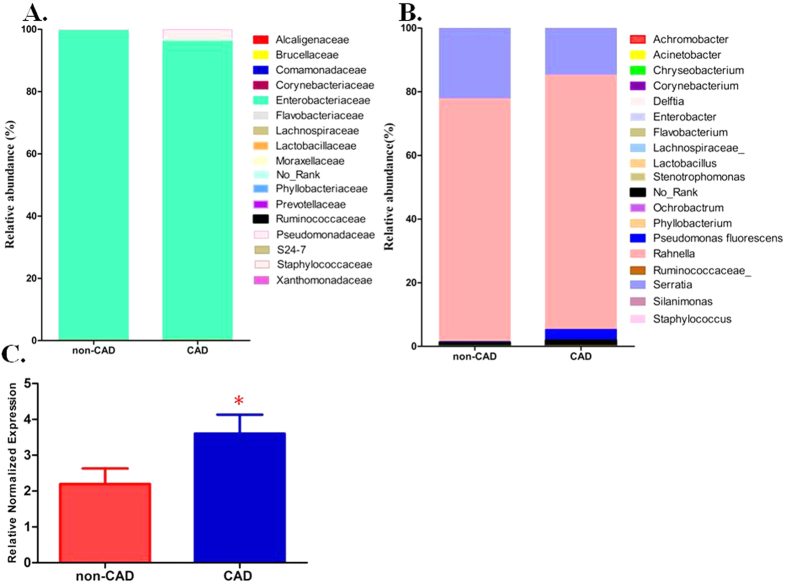
Bacterial taxa identified in blood DNA samples from CAD and non-CAD patients. (**A**,**B**) Relative abundance of taxonomic summary of the blood microbiota of CAD patients and non-CAD controls as determined by 16S rRNA gene pyrosequencing at family level (**A**) and genus level (**B**). (**C**) Quantification of the *Pseudomonas* 16S rRNA gene expression in bacteria positive samples from CAD patients (n = 53) and non-CAD patients (n = 31). The *Pseudomonas* 16s rRNA gene levels were normalized by the expression of bacterial 16s rRNA gene using the general primers targeting bacterial V1-V3 region. The data are expressed as mean ± SD. *P < 0.05 vs. non-CAD patients.

The 16S rRNA gene amplification from the blood sample indentified a diversity of bacteria at the family level, most detected taxa (8,824/9,203 in the CAD group, 9,009/9,064 in the non-CAD group) belonged to family *Enterobacteriaceaes*. The family *Pseudomonadaceae*, *Brucellaceae* and *Xanthomonadaceae* can also be detected in the sample. The members of family *Pseudomonadaceae* were more frequently identified in the CAD group (297/9203, 3.2%) than the non-CAD group (15/9064, 0.2%) (Fig. 1A, Supplementary Table 2). At the genus level, including some known bacterial taxa previous reported at the atherosclerotic plaque are also detected in our study, the most abundant was *Rahnella* (7353/9203 in the CAD group, 6912/9064 in the non-CAD group), *Serratia* and *Pseudomonas* can also be detected in the two groups, which were similar with previous studies reported the bacterial DNA in atherosclerotic plaques^8^ (Fig. 1B).

### Real-time PCR amplification of *Pseudomonas* in blood samples

In order to confirm the pyrosequencing results, we used species specific real-time PCR to quantify the expression of 16S rRNA gene of *Pseudomonas.* And universal primers for bacterial 16S rRNA gene PCR amplification was carried out to detect the presence of bacteria in blood samples. This study included a total of 206 patients suspected to suffer CAD in Daping hospital. Demographic data of the two study groups are presented in Table 1. In the present study, 102 blood DNA samples from 206 patients were positive for bacteria by using universal bacteria primers (49.5%). In the PCR analysis, the *Pseudomonas* sequence of the 16S rRNA gene can be detected in 84 patients, including 53 CAD patients and 31 non-CAD patients. In addition, *Pseudomonas* quantifications based on the 16S rRNA gene was significant higher in the CAD groups than the non-CAD group (Fig. 1C).

### Increased circulating zonulin levels in CAD patients

We attempted to understand how the bacteria enters the circulation and be detected in the blood. Most of the detected bacteria belonged to family *Enterobacteriaceaes*. In normal physiological conditions, the compact intestinal epithelium forms barriers to prevent the bacteria from invading the intestinal capillary. Zonulin reversibly modulate IP by regulating intestinal tight junctions. We measured the circulating zonulin levels by ELISA method. In the 206 patients, as a whole circulating zonulin was significantly higher in CAD patients (n = 126) versus non-CAD (n = 80) subjects (7.3 ± 1.8 vs 4.0 ± 1.6 ng/ml, P = 0.009) (Table 1).

### Enteric bacteria exposure increase intracellular zonulin levels

**Figure 2:**
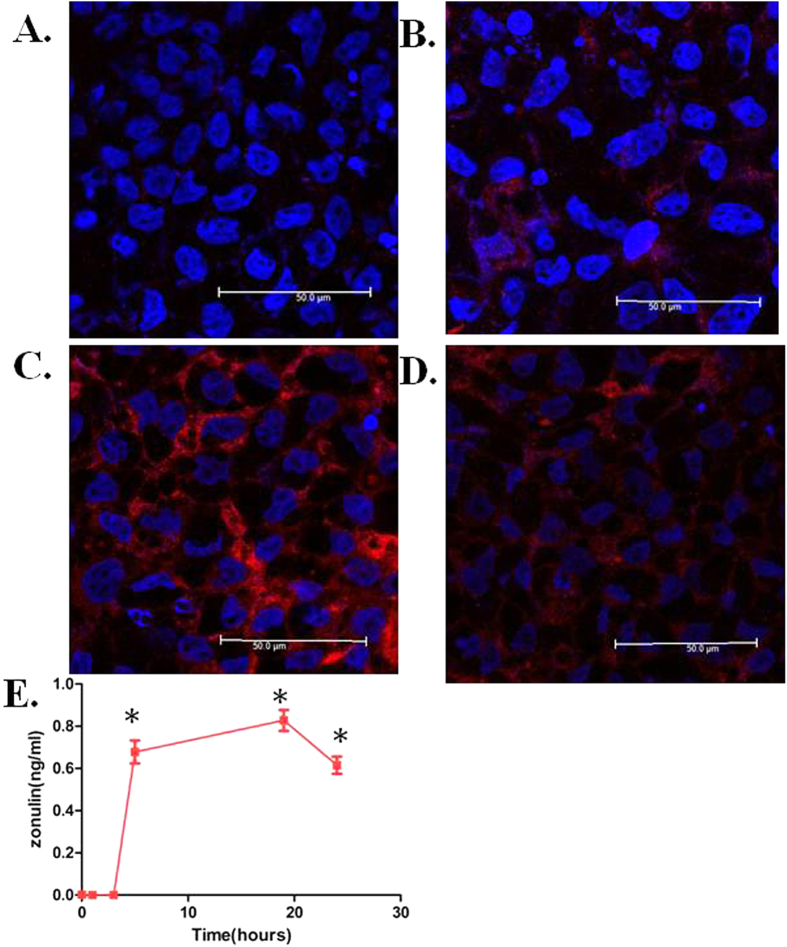
Effects of *Pseudomonas fluorescens* on zonulin expression in Caco-2 cells. (**A**) Human Caco-2 cell monolayer was exposed to *Pseudomonas fluorescens* and fixed with paraformaldehyde. Zonulin expression was determined by immunostaining with anti-zonulin antibodies (red), nucleus was stained by DAPI (blue). At 0 hour, the uninfected Caco-2 monolayer showed less zonulin staining in the cell cytoplasma. (**B**) One hour after *Pseudomonas fluorescens* exposure, zonulin staining can be visualized in the cytoplasma. (**C**) After 5 hrs of *Pseudomonas fluorescens* exposure, the area with zonulin staining full filled the entire cell cytoplasm indicate significant increase in zonulin expression. (**D**) Zonulin staining in cell cytoplasm gradually decreased 24 hrs after *Pseudomonas fluorescens* exposure. (**E**) Secreted form of zonulin released from Caco2 monolayers exposed to *Pseudomonas fluoresce*. The zonulin levels 0–5 hrs post-incubation in the cell culture supernatants were lower than the detection limit. *Pseudomonas fluorescens* induced significant zonulin release 5 hrs post-incubation and gradually increase at 19 hrs after bacterial exposure. The zonulin level decreased after 24 hrs of *Pseudomonas fluoresce* exposure. Data are expressed as mean ± SD (n = 3, *P < 0.05 versus 0 hour).

Considering the central role that zonulin plays in regulating intestinal tight junctions, it remains to be determined whether intestinal bacteria exposure could increase circulating zonulin levels. Previous studies have shown that intestinal cells deviate to the main source of zonulin secretion after bacterial exposure. An *in-vitro* intestinal barrier model was established by culturing the human colonic adenocarcinoma epithelial Caco-2 cell line in a transwell dish. Confocal immunofluorescence microscopy was used to analyze whether or not the bacterial colonization could affect the localization and expression of zonulin in the cell. Uninfected Caco-2 cells at 0 h showed only a little zonulin staining located at the cell periphery (Fig. 2A). One hour after adding the *Pseudomonas fluorescens* bacteria into the medium, the staining of intracellular zonulin gradually increased in the cytoplasm (Fig. 2B) and 5 hours (hrs) post-bacterial exposure, the zonulin rapidly increased and showed a fluorescent irregular pattern at the edge of the cells (Fig. 2C). The staining of intracellular zonulin gradually decreased after 24 hrs of bacterial exposure (Fig. 2D). The secreted form of zonulin, in spite of its being very low in the cell culture supernatants^13^, was slightly increased after 5 hrs of bacterial exposure and peaked at 19 hrs post-exposure as measured by ELISA (Fig. 2E).

### The increased zonulin expression by enteric bacteria increased IP in Caco-2 cell monolayer

**Figure 3:**
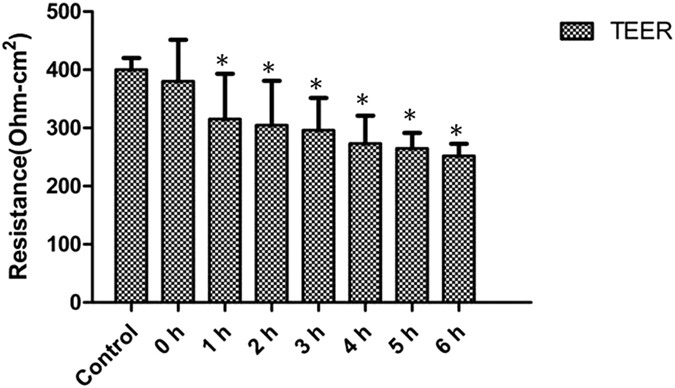
*Pseudomonas fluorescens* increased Caco-2 cell monolayer IP in a time-dependent manner. Caco-2 cell monolayers were cultured in transwells. A equal volume of *Pseudomonas fluorescens* suspension or PBS (control) were added to the upper chamber of transwell assay. TEER was monitored using a dual planar electrode for 6 hrs. The TEER decreases started from 1 hour post incubation compared to control monolayer and gradually decrease in a time- dependent manner. Data are expressed as mean ± standard error (n = 3, *P < 0.05 versus control group).

Due to the higher plasma zonulin levels in CAD patients, we next sought to investigate whether the increased zonulin expression results in higher IP. The Caco-2 monolayer was established to investigate the effects by measuring the TEER values. After 21 days of culturing, the Caco-2 cell monolayer fully covered the transwell membrane. The TEER was significantly higher in Caco-2 cell monolayer model (400 Ω · cm^2^) than in the medium (10 Ω · cm^2^), indicating the successful establishment of intestinal barrier model. *Pseudomonas fluorescens* were added into the upper medium of Caco2 monolayers and co-incubated, TEER were monitored both at baseline and after *Pseudomonas fluorescens* exposure. The *Pseudomonas fluorescens* addition induced a significant decrease in TEER in a time-dependent manner (Fig. 3). The inhibitory effect of *Pseudomonas fluorescens* on TEER rapidly reduced to 315 Ω · cm^2^ within one hour, and gradually decreased in a time-dependent manner, indicating that enteric bacteria exposure could increase IP in the Caco-2 cell monolayer model.

### Zonulin facilitated enteric bacteria penetration

**Figure 4:**
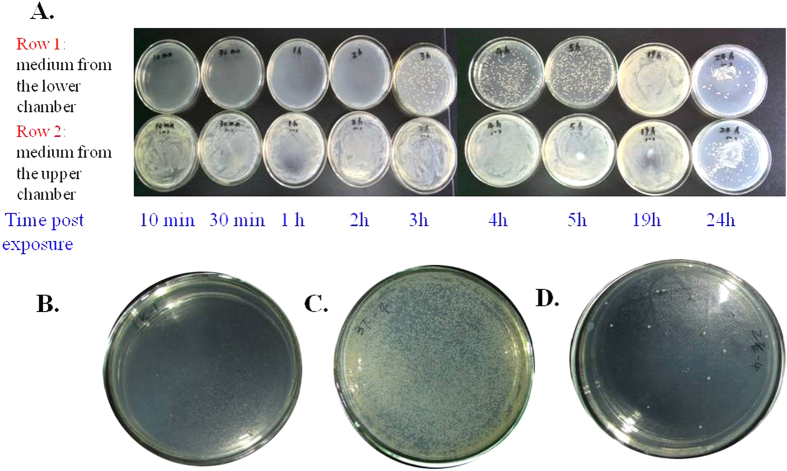
Bacteria culture of medium from lower chamber of Caco-2 cell transwell assay. (**A**,**B**) In the Caco-2 transwell assay, after adding the *Pseudomonas fluorescens* suspension (**A**) or PBS (**B**) to the upper chamber of transwell assay for 6 hrs, the medium in the lower culture chamber was taken, and cultured in a separate HE agar plate for another 24 hrs. The bacterial colonies in the plate was yellow and recorded by photography, the representative image is shown. (**C**) The medium in the upper chamber was removed from the transwell, the Caco-2 cell monolayer was trypsinized and washed twice with sterile PBS. The re-suspended cells were then plated onto HE agar plate and cultured for 24 hrs. The bacterial colonies in the plate was recorded by photography. (**D**) The medium in the lower chamber of the transwell units was collected at increasing time intervals (10 min, 30 min, 1 h, 2 h, 3 h, 4 h, 5 h, 19 h, 24 h) after adding *Pseudomonas fluorescens* to the upper chamber. The medium in the upper chamber of the transwell was collected at the corresponding time and then plated onto HE agar plate and cultured for 24 hrs (row 1: medium from the lower chamber, row 2: medium from the upper chamber).

**Figure 5:**
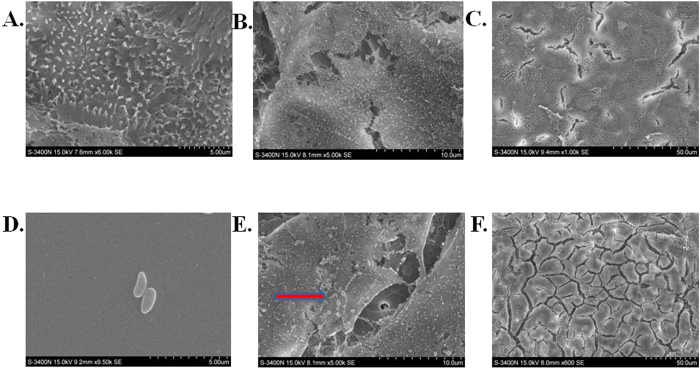
*Pseudomonas fluorescens* exposure induced morphological changes in Caco-2 cell monolayer. (**A**–**C**) The Caco-2 cell monolayer before *Pseudomonas fluorescens* exposure. The morphology was examined by transmission electron microscopy (TEM). The microvilli were tightly aligned (bar marker, 5 μm) with typical intense inter-cellular junctions ((**B**) bar marker, 20 μm; (**C**) bar marker, 50 μm). (**D**) The typical morphology of *Pseudomonas fluorescens* was coccus-shaped (bar marker, 5 μm). (**E**) Coccus-shaped *Pseudomonas fluorescens* adhering to the Caco-2 cell surface (Arrow bars) (bar marker, 20 μm). (**F**) *Pseudomonas fluorescens* exposure disrupted intercellular tight junctions, including extensive disorganization, augmentation, condensation, and beading of its normal intercellular structure. (bar marker, 50 μm).

As *Pseudomonas fluorescens* exposure significantly increase IP, we wonder whether the increased IP could facilitate bacteria penetration from the intestine. This hypothesis was challenged in the same transwell cell culture model as shown in Fig. 3. After 3 hrs of *Pseudomonas fluorescens* exposure in the upper chamber, the bacterial colonies can be visually detected by culturing the medium collected in the lower chamber of transwell assay in HE agar plate. The number of penetrated *Pseudomonas fluorescens* increased with the time of bacterial exposure as determined by increased number of bacterial colonies collected from different times after *Pseudomonas fluorescens* exposure. (Fig. 4A). No visible bacterial colonies were cultured in the medium from the lower chamber after adding the PBS control to Caco-2 monolayer (Fig. 4B). The Caco-2 monolayer cells were then subsequently trypsinized and re-suspended. The cells were cultured for an additional 24 hrs, but only a few bacteria could be detected in the plate, suggesting that most of the bacteria pass through the intercellular tight junctions but not phagocytized and reside in the Caco-2 cells (Fig. 4C). The bacteria consistently stimulated zonulin expression and secretion (Fig. 2E), resulting in higher IP. Zonulin released by Caco-2 cells reversibly led to the disassembly of intercellular tight junctions and then further enhancing more bacteria penetrate the intestinal epithelium in a time dependent manner. We also observed morphological changes in the Caco-2 cell microstructure after bacterial exposure. Transmission electron micrographs revealed that coccus-shaped bacteria entangled in the Caco-2 monolayer. *Pseudomonas fluorescens* were observed to be adhered to the microvilli cell surface (Fig. 5A–E). Moreover, the intercellular junction of Caco-2 cell monolayer was enlarged after *Pseudomonas fluorescens* exposure (Fig. 5F).

## Discussion

The role of bacteria in atherosclerosis progression have been extensively studied for more than two decades. Low levels of bacteria could be found in the circulation in many chronic metabolic diseases, including obesity, type 2 diabetes and atherosclerosis, and is commonly referred to as “metabolic endotoxemia”^14^. For example, previous studies suggested that chronic bacterial infection, eg. periodontitis and *Chlamydia pneumoniae* were clinically associated with atherosclerotic diseases, which therefore was taken as an additional risk factor^4^. Our present study found that in CAD patients without symptomatic signs of any infection, the presence of a diversity of bacteria in the blood was confirmed by 16S rRNA gene amplification, most belong to *Enterobacteriaceaes*. Zonulin was found to be significantly higher in CAD patients. *In-vitro* experimental showed that the enteric derived bacteria *Pseudomonas fluorescens* increased zonulin expression and secretion in Caco-2 cells and consequently increased IP, leading to more *Pseudomonas fluorescens* penetration of the transwell membrane. Our results indicated that the elevated zonulin in atherosclerosis may increase intestine IP and facilitate transportation of bacteria from the intestine to the blood.

The mechanisms, regarding the contribution of bacteria in atherosclerosis, are complicated. Previous studies mainly focused on the indirect mechanisms. Microbial activation of these innate immune receptors promotes inflammation that dampens reverse cholesterol transport, which in turn augments insulin resistance, hyperlipidemia, and vascular inflammation. Recent studies revealed that, in addition to digestion and absorption of many nutrients, intestinal microbial flora may play an active role in the development of complex metabolic disease by the production of metabolites^6^,^7^,^15^. Our results revealed that most of the bacterial species in the blood belong to *Enterobacteriaceaes.* The opportunistic pathogens, *Rahnella* and *Serratia* which have been directly linked to human diseases in rare instances, are the most abundant genus^16^,^17^,^18^. Their presence in the blood in our study raises the possibility that they may directly affect the pathogenesis of atherosclerosis. In contrast to previous studies, we did not detect reported bacteria in other published papers, eg. *Chlamydia pneumoniae*, *Helicobacter*, *Veillonella* and *Streptococcus*^6^. Our result is consistent with Armingohar’s study, which reported a relatively high percentage of the16S rRNA genes detected in vascular biopsies from patients with chronic periodontitis belonged to the *Enterobacteriaceae* (*Serratia* and *Klebsiella*)^8^. The discrepancy between our reports with previous studies may be related to the sample, race or even different dietary habits. In addition, variations associated with DNA extraction method and biological variations are also a contributor to different microbiota profiles^19^.

Previous studies indicated that intestinal bacteria induced changes in zonulin secretion and IP in an “on and off” manner, irrespective of the bacteria species the intestine challenged^13^. *Rahnella aquatilis* is a Gram negative environment microorganism first isolated in the water. Whether or not they have the ability to infect the body depends on the host’s immunological state. However, our enrolled patients were all free of immune deficient diseases and have no symptoms of acute or chronic gastroenteritis. It is more likely that small amounts of enteric bacteria enter into the blood circulation in a chronic process without inducing acute inflammatory responses.

The intact intestinal epithelium is the largest mucosal surface that separates the intestinal microbiota from the host circulation in physiological conditions. However, under pathological conditions, the dysfunction of the intestinal barrier function increases IP and contributes to the development of systemic inflammatory response syndrome in seriously sick patients^20^. The cytokines, generated in the gut epithelium during systematic or localized inflammation, could increase IP and facilitate intestinal bacterial translocation^21^. When the bacteria are phagocytized by immunocytes, leading to more cytokine release, this could generate a vicious cycle^22^. Proper function of IP is crucial to sustain the intact barrier and maintain normal physiological processes. The tight junction is made up of several strands wrapping around the circumference of the apical portion of the intestinal epithelial cells. Zonulin physiological regulate IP reversability by modulating the epithelial tight junctions^23^. Our studies revealed that zonulin was significantly elevated in CAD patients. The exposure of Caco-2 cell lines to *Pseudomonas fluorescens* increase zonulin expression. Zonulin induces actin polymerization, followed by cytoskeleton redistribution to the subcortical cell compartment. Zonulin also induces cytoskeletal changes, followed by tight junction disassembly, resulting in increased intestinal para-cellular permeability^23^. The detection of intestinal bacterial by 16S rRNA gene amplification in both the blood sample and atherosclerotic plaques supports the notion that intestinal bacteria in plaques may original from the intestine.

In our results, only a few bacteria could be detected in the Caco-2 cells in the transwell, suggesting that the bacteria pass the intestinal barrier through a intercellular way. However, the mechanism by which the enteric bacteria could induce zonulin secretion and disturb the intestinal tight-junction permeability under pathological conditions remains unclear. Although bacteria may play an important role in the development of atherosclerotic disease, several large randomized control trials failed to prove that utilizing antibiotics targeted against *Chlamydia pneumoniae* to suppress the microbiota could improve the clinical outcomes^24^,^25^. Bacteria may act in a “hit and run” role, meaning that bacterial infection may initiate the atherosclerosis in the early stages but not during the active part of the progression of disease. The gut microbiota transplantation can transmit the atherosclerosis susceptibility, therefore, it is possible to reconstruct more healthy gut bacteria flora to alleviate CAD development^26^. Given the fact that zonulin is significantly elevated in atherosclerosis and increase intestinal IP, it’s possible that targeting zonulin using monoclonal antibody or inhibitors maybe a provocatively new way to move forward in CAD prevention and treatment.

The major limitations of the study was that we have not established a *in vivo* model to test our hypothesis. Which cells were responsible to carry the bacteria in the circulation and how to transfer them to the plaque requires further investigation. Moreover, other requirements especially the inflammatory stimulation are required for leading to increased IP and dissemination of gut bacteria into circulation^27^, and this were not reflected in our *in vitro* model. Furthermore, plaque vulnerability plays an important role in the coronary events^28^, whether the elevated zonulin increase bacterial load and activate immune cells in patients with acute coronary syndrome and increase plaque vulnerability remains further investigation.

## Methods

### Subjects

A total of 8 angiography confirmed patients with CAD and 8 non-CAD patients were recruited into the study from the Department of Cardiology in Daping Hospital between March to July in 2012. Another cohort comprised of 206 suspected CAD patients were recruited from Daping Hospital in April 2016. Patients with active infectious disease or gastroenteritis were excluded. Anthropometric data were collected at the time of recruitment. Blood samples were collected after 12 hrs of fasting; plasma was isolated by centrifugation at 1,800× g for 15 min. The diagnostic criterion for CAD was the patient having more than a 50% luminal diameter narrowing of the vessel. A written informed consent for this study was obtained from all the participants. All study protocols were in accordance with the Declaration of Helsinki and were approved by the Medical Ethics Committee of Daping Hospital.

### ELISA detection of zonulin

The zonulin concentrations in plasma and cell culture supernatants were measured in duplicate using an ELISA kit (Immundiagnostik AG, Germany) according to the manufacturer’s instructions^10^. The absorbance at 450 nm was measured with a microplate auto-reader (Varioskan Flash, Thermo Fisher Scientific, USA). The lower limit of detection for this assay is 0.225 ng/ml.

### Microbial diversity analysis

#### DNA extraction and PCR amplification

A 5 ml sample of blood from each subject was collected into a tube containing ethylenediamine tetra-acetic acid (EDTA) as an anticoagulant. The blood DNA extraction was performed using the TIANamp Genomic DNA Kit (Tiangen Biotech, China), according to the manufacturer’s recommendations.

#### 454 pyrosequencing and processing of pyrosequencing data

After purification using the AxyPrep DNA Gel Extraction Kit (Axygen Biosciences, U.S.) and quantification using QuantiFluor-ST (Promega, U.S.), a mixture of amplicons was used for pyrosequencing on a Roche 454 GS FLX+ Titanium platform (Roche 454 Life Sciences, U.S.) according to standard protocols^29^. The resulting sequences were processed using Seqcln (http://sourceforge.net/projects/seqclean/) and Mothur. After removing low quality sequences (<Q25) and sequences shorter than 200 bp, with homopolymers longer than six nucleotides, and containing ambiguous base calls or incorrect primer sequences, a total of 18,267 high-quality sequences were produced with an average length of 500 bp per sequence. Sequences were aligned against the silva database (http://www.arb-silva.de/) using k-mer searching (http://www.mothur.org/wiki/Align.seqs). Potentially chimeric sequences were detected using UCHIME (http://drive5.com/uchime) and removed. The remaining reads were pre-clustered (http://www.mothur.org/wiki/Pre.cluster) and then clustered using uncorrected pairwise algorithm. In addition, Operational taxonomic units were defined as sharing >97% sequence identity using Furthest neighbor method (http://www.mothur.org/wiki/Cluster).

### Quantification of *Pseudomonas* by quantitative PCR

Species specific real-time PCR were applied for quantifying the *Pseudomonas* in blood using previous reported primers targeting *Pseudomonas* 16S rRNA gene located in the V3-V4 hypervariable region^30^. The negative control was sterile distilled water instead of template DNA and were included in each batch of samples. Real-time quantification was performed using SYBR premix Ex TaqTM II according to the manufacturer’s recommendations (TaKaRa, Japan). Twenty microliters of final reaction mixture contained 10 ul of Ex Taq, 1 ul of sense primer, 1 ul of antisense primer, 7 ul of sterile deionized water, and 1 ul of DNA extract. Thermocycling was conducted using a CFX96 Real-Time PCR Detection System (Bio-Rad, U.S.) initiated by a 30 sec incubation at 95 °C, followed by 35 cycles at 94 °C for 5 sec and 60 °C for 45 sec with a single fluorescent reading taken at the end of each cycle. Each reaction was conducted in triplicate. All the runs were completed with a melt curve analysis to confirm the specificity of amplification and lack of primer dimers. Samples with a CT equal or above the negative control were treated as below the limit of detection. PCR products were analyzed by agarose gel electrophoresis. The V1-V3 region of the bacterial 16S rRNA gene was also quantified using universal primers and used at a ratio Pseudomonas 16S rRNA gene /16S rRNA gene^8^.

### Cell culture

Human colon adenocarcinoma-derived cells (Caco-2) were purchased from the Institute of Biochemistry and Cell Biology of the Chinese Academy of Sciences (Shanghai, China). The cell lines were cultured in Dulbecco’s Modified Eagle Medium (DMEM, Gibco, U.S.), containing 10% fetal bovine serum (FBS, Gibco, U.S.) as well as 100 U/ml penicillin and 100 μg/ml streptomycin (Invitrogen , U.S.). Cells were maintained in a humidified incubator at 37 °C in the presence of 5% CO_2_.

### Bacteria culture

*Pseudomonas fluorescens* were obtained from the CCTCC (China Center for Type Culture Collection, Wuhan, China). The bacteria were grown on luria broth (LB) agar overnight at 37 °C from frozen stocks. A single colony was transferred to a brain heart infusion (BHI) broth and incubated for 3–5 days at 37 °C. Bacteria were harvested from the broth culture by centrifugation at 2,500 g for 15 min and washed twice with sterile phosphate buffered solution (PBS) solution. The bacteria were then re-suspended in PBS to a final concentration of 10^9^ CFU/ml by optical density measurement.

### Intestinal permeability study in the transwell system

Caco-2 monolayer cells (5 × 10^4^ cells/ml) were applied into transwell inserts (PET Clear membrane; area, 0.6 cm^2^; pore size, 1.0 μm; Millicell, Millipore, Germany). Cells were cultured in complete media with hydrolysates concentration of 0.05 g/L. The medium was added to both lower and upper chambers of the transwell inserts, with the volumes kept at 100 and 600 μL, respectively. Cells were fed with fresh medium every other day for 2 weeks. Each treatment contained six transwell inserts. Twelve hrs before the experimental assays were performed; the medium of the transwell inserts was replaced with a medium devoid of antibiotics.

The medium in the transwell inserts was withdrawn; and 10 μL of the *Pseudomonas fluorescens* suspension was applied to the apical surface of the Caco-2 cells, equal volume of PBS solution was taken as control. Fresh antibiotic-free medium of 100 μL was added to the upper chamber, and 600 μL of fresh antibiotic-free medium was added to the lower chamber. The cell monolayer was gently agitated for 10 min before they were incubated for 3 hrs. In this experiment, six transwell inserts were assigned to each treatment group. The medium was withdrawn from the lower and upper chambers of the transwell units and plated onto HE agar plates either directly or after 10-fold serial dilution in sterile PBS from 10 min to 24 hrs after bacterial exposure. Plates were incubated at 37 °C for 24 hrs, at which time the appearance of bacterial colonies were recorded by photography. *Pseudomonas fluorescens* appeared as yellow colonies, it did not secrete zonulin in their culture supernatants, as established by ELISA.

### Transepithelial electrical resistance measurements

Intestinal permeability was assessed *in vitro* by measuring trans-epithelial electrical resistance (TEER) of using a dual planar electrode, expressed in Ω · cm^2^ and normalized by the baseline resistance values. The whole process was carried out at a constant temperature (37 °C). Three areas with different directions at each transwell were selected and TEER was measured three times for each area.

### Transmission electron microscopy

The Caco-2 cell monolayer was washed three times with 0.1 mol/L sterile PBS and fixed overnight at 4 °C in 2.5% glutaraldrhyde solution. The samples were added into cold 0.1 mol/L PBS with 1% osmium tetroxide for 60 min at room temperature, and then rinsed twice with 0.1 mol/L PBS. Samples were dehydrated through graded ethanol series for 10 min per step and embedded in Spurr’s epoxy resin. Samples were sectioned and stained with uranyl acetate and lead citrate and observed using a Hitachi-7500 transmission electron microscope (Hitachi, Japan).

### Localization of zonulin by confocal immunofluorescence microscopy

A monolayer of Caco-2 cells was seeded onto glass cover slips in a 24-well plate at a density of 2 × 10^6^ cells and grown to 50% to 60% confluence. The cells were then similarly exposed to *Pseudomonas fluorescens* as previously described. Subsequently, the cells were fixed in 1% paraformaldehyde, permeabilized with 0.2% Triton X-100, and washed in PBS. Mouse monoclonal antibody against human zonulin (1:50 dilution, Abcam, U.S.) was added to the cells and incubated for more than 12 hrs at 4 °C. After incubation with rhodamine-conjugated goat anti-mouse IgG (1:200 dilution) for 30 min, the cells were washed again with PBS. Nuclei were stained with 1 μg/mL DAPI for 3 min. The localization of zonulin in the cells was examined under an Axiovert 40 microscope (Carl Zeiss, Germany).

### Statistical analyses

The data were presented as means and standard deviations unless otherwise stated. Continuous variables were compared using one-way ANOVA followed by Dunnetts’s post-hoc test, and categorical variables were compared using the χ2 test using the Statistical Package for Social Sciences software package (SPSS Statistics version 18.0, U.S.). Mann-whitney U test was used to compare the data with partial distribution. All reported p values are 2-tailed and were considered statistically significant at <0.05.

## Additional Information

**How to cite this article**: Li, C. *et al.* Zonulin Regulates Intestinal Permeability and Facilitates Enteric Bacteria Permeation in Coronary Artery Disease. *Sci. Rep.* **6**, 29142; doi: 10.1038/srep29142 (2016).

## Supplementary Material

Supplementary Information

## Figures and Tables

**Table 1:** Demographic data of patients with or without CAD.

	CAD group (n = 126)	Non-CAD group (n = 80)	P value
N (M/F)	65/61	36/44	0.319
Age (year)	63.5 ± 11.1	62.1 ± 13.2	0.521
BMI (kg/m^2^)	23.7 ± 3.5	23.5 ± 3.6	0.738
SBP(mmHg)	125.2 ± 17.6	121.6 ± 16.4	0.136
DBP(mmHg)	74.0 ± 9.2	73.3 ± 8.9	0.623
Total cholesterol (mmol/l)	4.6 ± 1.0	4.4 ± 0.9	0.488
Triglyceride (mmol/l)	1.5(1.0–2.3)	1.2(0.9–1.9)	0.029#
LDL-C (mmol/l)	2.8 ± 0.7	2.7 ± 0.6	0.569
HDL-C (mmol/l)	1.1 ± 0.3	1.2 ± 0.3	0.088
Hypertension (%)	57.1	45.0	0.115
Diabetes (%)	23.8	15	0.156
Zonulin(ng/ml)	7.3 ± 1.8	4.0 ± 1.6	0.009

^#^Data are presented as median (interquartile range) and compared by Mann-whitney U test.
