# Supplementary material for: Zonulin Regulates Intestinal Permeability and Facilitates Enteric Bacteria Permeation in Coronary Artery Disease
Source: Sci Rep. 2016 Jun 29;6:29142. doi: 10.1038/srep29142 (PMC4926221; doi:10.1038/srep29142)
Supplement: Supplementary Information [file srep29142-s1.doc]

**Zonulin Regulates Intestinal Permeability and Facilitates Enteric Bacteria Permeation in Coronary Artery Disease**

Chuanwei Li^1,4#^, Min Gao^2#^, Wen Zhang^3^, Caiyu Chen^1,4^, Faying Zhou^1,4^, Zhangxu Hu^3^*, Chunyu Zeng^1,4^*

**Supplementary Table 1. Demographic data of patients with or without CAD in 454 pyrosequencing analysis**

|  | ***CAD group（n=8）*** | ***Non-CAD group（n=8）*** | ***P value*** |
| --- | --- | --- | --- |
| **N (M/F)** | **4/4** | **4/4** | **1** |
| **Age (year)** | **64.9±10.1** | **63.8±9.8** | **0.875** |
| **BMI (kg/m^2^)** | **24.5±2.8** | **23.8±3.0** | **0.842** |
| **Total cholesterol (mmol/l)** | **5.20±0.95** | **4.87±0.89** | **0.214** |
| **Triglyceride (mmol/l)** | **1.76±1.17** | **1.53±1.10** | **0.324** |
| **LDL-C (mmol/l)** | **2.74±0.59** | **2.47±0.53** | **0.326** |
| **HDL-C (mmol/l)** | **1.42±0.34** | **1.35±0.38** | **0.452** |
| **Hypertension (%)** | **62.5%** | **50.0%** | **0.614** |
| **Diabetes (%)** | **37.5%** | **25.0%** | **0.59** |
| **Dyslipidemia (%)** | **50.0%** | **37.5%** | **0.782** |
| **Current Smokers (%)** | **25.0%** | **25.0%** | **1** |
| **Medication** |  |  |  |
| **Statin (%)** | **100%** | **100%** | **1** |
| **Aspirin (%)** | **100%** | **100%** | **1** |
| **ACEI/ARB (%)** | **50%** | **37.5%** | **0.614** |

**Supplementary Table 2. Bacterial taxa identified in mixed blood DNA samples from patients with CAD and non-CAD patients**

| **Groups at the** **phylum level** | **Non-CAD group** | **CAD**  **group** | **P value** |
| --- | --- | --- | --- |
| Actinobacteria{phylum} | 1 | 0 | 0.312 |
| Bacteroidetes{phylum} | 4 | 2 | 0.399 |
| Cyanobacteria{phylum} | 0 | 3 | 0.087 |
| Firmicutes{phylum} | 4 | 2 | 0.399 |
| Proteobacteria{phylum} | 9055 | 9196 | 0.764 |
| All (16S rRNA sequence frequency) | 9064 | 9203 |  |

| **Groups at the class level** | **Non-CAD group** | **CAD**  **group** | **P value** |
| --- | --- | --- | --- |
| Actinobacteria(class){class} | 1 | 0 | 0.312 |
| Alphaproteobacteria{class} | 9 | 10 | 0.856 |
| Bacilli{class} | 2 | 0 | 0.153 |
| Bacteroidia{class} | 3 | 1 | 0.307 |
| Betaproteobacteria{class} | 4 | 4 | 0.976 |
| Chloroplast{class} | 0 | 3 | 0.087 |
| Clostridia{class} | 2 | 2 | 0.983 |
| Flavobacteria{class} | 1 | 1 | 0.988 |
| Gammaproteobacteria{class} | 9042 | 9182 | 0.761 |
| All (16S rRNA sequence frequency) | 9064 | 9203 |  |

| **Groups at the order level** | **Non-CAD group** | **CAD**  **group** | **P value** |
| --- | --- | --- | --- |
| Bacilli_Bacillales{order} | 1 | 0 | 0.312 |
| Bacilli_Lactobacillales{order} | 1 | 0 | 0.312 |
| Bacteroidales{order} | 3 | 1 | 0.307 |
| Burkholderiales{order} | 4 | 4 | 0.976 |
| Clostridiales{order} | 2 | 2 | 0.983 |
| Corynebacteriales{order} | 1 | 0 | 0.312 |
| **Enterobacteriales{order}** | **9009** | **8824** | **0.044** |
| Flavobacteriales{order} | 1 | 1 | 0.988 |
| No_Rank | 3 | 34 | <0.000 |
| **Pseudomonadales{order}** | **22** | **324** | <0.000 |
| Rhizobiales{order} | 9 | 10 | 0.856 |
| Xanthomonadales{order} | 8 | 3 | 0.123 |
| All (16S rRNA sequence frequency) | 9064 | 9203 |  |

| **Groups at the family level** | **Non-CAD group** | **CAD**  **group** | **P value** |
| --- | --- | --- | --- |
| Alcaligenaceae{family} | 3 | 4 | 0.727 |
| Brucellaceae{family} | 8 | 10 | 0.671 |
| Comamonadaceae{family} | 1 | 0 | 0.312 |
| Corynebacteriaceae{family} | 1 | 0 | 0.312 |
| **Enterobacteriaceae{family}** | **9009** | **8824** | **0.044** |
| Flavobacteriaceae{family} | 1 | 1 | 0.988 |
| Lachnospiraceae{family} | 2 | 1 | 0.551 |
| Lactobacillaceae{family} | 1 | 0 | 0.312 |
| Moraxellaceae{family} | 0 | 7 | 0.009 |
| No_Rank | 10 | 54 | <0.000 |
| Phyllobacteriaceae{family} | 1 | 0 | 0.312 |
| Prevotellaceae{family} | 3 | 0 | 0.08 |
| **Pseudomonadaceae{family}** | **15** | **297** | **<0.000** |
| Ruminococcaceae{family} | 0 | 1 | 0.323 |
| S24-7{family} | 0 | 1 | 0.323 |
| Staphylococcaceae{family} | 1 | 0 | 0.312 |
| Xanthomonadaceae{family} | 8 | 3 | 0.123 |
| All (16S rRNA sequence frequency) | 9064 | 9203 |  |

| **Groups at the genus level** | **Non-CAD group** | **CAD**  **group** | **P value** |
| --- | --- | --- | --- |
| Achromobacter{genus} | 3 | 4 | 0.727 |
| Acinetobacter{genus} | 0 | 7 | 0.009 |
| Chryseobacterium{genus} | 1 | 0 | 0.312 |
| Corynebacterium{genus} | 1 | 0 | 0.312 |
| Delftia{genus} | 1 | 0 | 0.312 |
| Enterobacter{genus} | 3 | 0 | 0.08 |
| Flavobacterium{genus} | 0 | 1 | 0.323 |
| Lachnospiraceae_{genus} | 0 | 1 | 0.323 |
| Lactobacillus{genus} | 1 | 0 | 0.312 |
| No_Rank | 98 | 164 | <0.000 |
| Ochrobactrum{genus} | 8 | 10 | 0.671 |
| Phyllobacterium{genus} | 1 | 0 | 0.312 |
| **Pseudomonas fluorescens{genus}** | **15** | **297** | **<0.000** |
| **Rahnella{genus}** | **6912** | **7353** | **0.072** |
| Ruminococcaceae_{genus} | 0 | 1 | 0.323 |
| **Serratia{genus}** | **2011** | **1362** | **<0.000** |
| Silanimonas{genus} | 1 | 0 | 0.312 |
| Staphylococcus{genus} | 1 | 0 | 0.312 |
| Stenotrophomonas{genus} | 7 | 3 | 0.194 |
| All (16S rRNA sequence frequency) | 9064 | 9203 |  |
